# Histoplasmosis in Persons Living with HIV

**DOI:** 10.3390/jof6010003

**Published:** 2019-12-18

**Authors:** Mathieu Nacher

**Affiliations:** 1 Centre d’Investigation Clinique Antilles Guyane, Inserm CIC1424, Centre Hospitalier de Cayenne, 97300 Cayenne, French Guiana; mathieu.nacher66@gmail.com; 2 EA3593 Ecosystèmes Amazoniens et Pathologie Tropicale (EPaT), Université de Guyane, DFR Santé, 97300 Cayenne, French Guiana

The increase in the number of immunocompromised persons, following the HIV pandemic, has led to a dramatic amplification of the number of patients with progressive disseminated histoplasmosis. Other causes of immune suppression linked to therapeutic immune suppression are increasingly frequent. For HIV-infected patients, disseminated histoplasmosis has been an AIDS-defining infection since 1987, and in the USA, for the past three decades, it has been relatively easily to diagnose with antigen detection tests [1]. However, elsewhere in endemic regions for histoplasmosis, the disease is still often unknown and undiagnosed, because simple diagnostic tests are unavailable. Thus, in most endemic areas, in HIV-infected patients, progressive disseminated histoplasmosis is often mistaken for its main differential diagnosis—miliary tuberculosis [2]. This lack of awareness of histoplasmosis is estimated to kill numerous patients every year in Latin America and beyond [3]. As new rapid diagnostic tests based on antigen detection will soon be commercially available, Latin American researchers have endeavored to increase awareness, and to build a diagnostic and treatment capacity throughout Latin America [4]. First it was 80% by 2020 targeted in Paramaribo [5], which has now been replaced by the Manaus declaration objective of 100% by 2025, whereby all hospitals should have a diagnostic and treatment capacity for histoplasmosis. In the present Issue, histoplasmosis has been approached from different perspectives. The different ways, clinicians, biologists, and researchers have become aware of the importance of histoplasmosis in different contexts, as follows: Terezinha Silva Leitao and colleagues present a focus article on their experience in Fortaleza Brazil, which shows the importance of the buffy coat direct examination and culture in the absence of antigen detection or PCR [6]. Pierre Couppié and colleagues share over 30 years of their experience, and show the wide range of clinical presentations that can lead to the diagnosis of disseminated histoplasmosis [7]—not only is it difficult to differentiate between disseminated histoplasmosis and miliary tuberculosis, but the two are frequently associated with very difficult diagnostic and therapeutic problems. Diego H Caceres and Audrey Valdes focus on the issue of the association of histoplasmosis and tuberculosis [8]. As the difficulties in diagnosing histoplasmosis have perpetuated a low awareness, Felix Bongomin and David Denning from GAFFI emphasize the need for advocacy with international Public Health Agencies in order to create conditions for the optimal diagnosis and treatment of this major killer of HIV-infected patients [9]. In the next few years, as new diagnostic tests are scaled up, and as the increased awareness momentum grows to fill the knowledge gaps, histoplasmosis may change from a confidential pathogen known by few, to a more “mainstream” public health problem with well-defined clinical algorithms everywhere. The past, and indeed the present, were/are riddled with difficulties, and this, in a way, is precisely what all of the articles the present series on histoplasmosis have in common. However, we may be reaching a crossroads, and these difficulties will hopefully soon be overcome with the new diagnostic tools and the light they will shed on non-specific infectious syndromes. Indeed, the realization that there is a significant untapped market in low- and middle-income countries should drive competition for the best and cheapest tests. Moreover, WHO/PAHO has seized the problem, and diagnostic and treatment guidelines are now being finalized, which is a source of great hope for all clinicians that do not have the proper tools to efficiently diagnose and treat their patients. In this context, the Manaus declaration of 100% by 2025 is an ambitious, but achievable goal, that should mobilize us all.

## References

[1] Wheat L.J., Freifeld A.G., Kleiman M.B., Baddley J.W., McKinsey D.S., Loyd J.E., Kauffman C.A. (2007). Clinical practice guidelines for the management of patients with histoplasmosis: 2007 update by the Infectious Diseases Society of America. Clin. Infect. Dis..

[2] Adenis A., Nacher M., Hanf M., Basurko C., Dufour J., Huber F., Aznar C., Carme B., Couppie P. (2014). Tuberculosis and histoplasmosis among human immunodeficiency virus-infected patients: A comparative study. Am. J. Trop. Med. Hyg..

[3] Adenis A.A., Valdes A., Cropet C., McCotter O.Z., Derado G., Couppie P., Chiller T., Nacher M. (2018). Burden of HIV-associated histoplasmosis compared with tuberculosis in Latin America: A modelling study. Lancet Infect. Dis..

[4] Neglected histoplasmosis in Latin America Group (2016). Disseminated histoplasmosis in Central and South America, the invisible elephant: The lethal blind spot of international health organizations. AIDS.

[5] Nacher M., Adenis A. (2016). Proceedings of First Histoplasmosis in the Americas and the Caribbean Meeting, Paramaribo, Suriname, December 4–6, 2015. Emerg. Infect. Dis..

[6] Leitão T.M.J.S., Oliveira Filho A.M.P., Sousa Filho J.E.P., Tavares B.M., Mesquita J.R.L., Farias L.A.B.G., Mota R.S., Nacher M., Damasceno L.S. (2019). Accuracy of Buffy Coat in the Diagnosis of Disseminated Histoplasmosis in AIDS-Patients in an Endemic Area of Brazil. J. Fungi.

[7] Couppié P., Herceg K., Bourne-Watrin M., Thomas V., Blanchet D., Alsibai K.D., Louvel D., Djossou F., Demar M., Blaizot R. (2019). The Broad Clinical Spectrum of Disseminated Histoplasmosis in HIV-Infected Patients: A 30 Years’ Experience in French Guiana. J. Fungi.

[8] Caceres D.H., Valdes A. (2019). Histoplasmosis and Tuberculosis Co-Occurrence in People with Advanced HIV. J. Fungi.

[9] Bongomin F., Kwizera R., Denning D.W. (2019). Getting Histoplasmosis on the Map of International Recommendations for Patients with Advanced HIV Disease. J. Fungi.

